# Radiomics-Derived Data by Contrast Enhanced Magnetic Resonance in RAS Mutations Detection in Colorectal Liver Metastases

**DOI:** 10.3390/cancers13030453

**Published:** 2021-01-25

**Authors:** Vincenza Granata, Roberta Fusco, Antonio Avallone, Alfonso De Stefano, Alessandro Ottaiano, Carolina Sbordone, Luca Brunese, Francesco Izzo, Antonella Petrillo

**Affiliations:** 1 Radiology Division, Istituto Nazionale Tumori—IRCCS—Fondazione G. Pascale, Via Mariano Semmola, 80121 Naples, Italy; v.granata@istitutotumori.na.it (V.G.); a.petrillo@istitutotumori.na.it (A.P.); 2 Abdominal Oncology Division, Istituto Nazionale Tumori—IRCCS—Fondazione G. Pascale, Via Mariano Semmola, 80121 Naples, Italy; a.avallone@istitutotumori.na.it (A.A.); a.destefano@istitutotumori.na.it (A.D.S.); a.ottaiano@istitutotumori.na.it (A.O.); 3 Radiology Division, Università degli Studi del Molise, Via Francesco De Sanctis, 86100 Campobasso, Italy; carolinasbordone2@gmail.com (C.S.); luca.brunese@unimol.it (L.B.); 4 Hepatobiliary Surgical Oncology Division, Istituto Nazionale Tumori—IRCCS—Fondazione G. Pascale, Via Mariano Semmola, 80121 Naples, Italy; f.izzo@istitutotumori.na.it

**Keywords:** radiomics, contrast enhanced magnetic resonance imaging, RAS mutation, colorectal liver metastases

## Abstract

**Simple Summary:**

In the present study, we assessed the association of RAS mutation status and radiomics derived data by Contrast Enhanced Magnetic Resonance Imaging (CE-MRI) in liver metastases by CRC. We performed the evaluation extracting by CE-MRI both texture and morphological metrics in a 3D setting. We demonstrated that radiomics with texture parameters could add value to qualitative assessment of MR studies and with better results compared to morphological metrics, providing individualized evaluation of CRLM. Texture parameters derived by CE-MRI and combined using multivariate analysis and patter recognition approaches could allow stratifying the patients according to RAS mutation status.

**Abstract:**

*Purpose*: To assess the association of RAS mutation status and radiomics-derived data by Contrast Enhanced-Magnetic Resonance Imaging (CE-MRI) in liver metastases. *Materials and Methods*: 76 patients (36 women and 40 men; 59 years of mean age and 36–80 years as range) were included in this retrospective study. Texture metrics and parameters based on lesion morphology were calculated. Per-patient univariate and multivariate analysis were made. Wilcoxon-Mann-Whitney U test, receiver operating characteristic (ROC) analysis, pattern recognition approaches with features selection approaches were considered. *Results*: Significant results were obtained for texture features while morphological parameters had not significant results to classify RAS mutation. The results showed that using a univariate analysis was not possible to discriminate accurately the RAS mutation status. Instead, considering a multivariate analysis and classification approaches, a KNN exclusively with texture parameters as predictors reached the best results (AUC of 0.84 and an accuracy of 76.9% with 90.0% of sensitivity and 67.8% of specificity on training set and an accuracy of 87.5% with 91.7% of sensitivity and 83.3% of specificity on external validation cohort). *Conclusions*: Texture parameters derived by CE-MRI and combined using multivariate analysis and patter recognition approaches could allow stratifying the patients according to RAS mutation status.

## 1. Introduction

Radiomics consists of the extraction of several parameters by radiological data that can provide information about tumor phenotype as well as the cancer microenvironment. Radiomics, when combined with other data linked to patient outcome, can produce precise evidence-based clinical-decision support systems (CDSS) [1]. The main task is to combine and to collect diverse multimodal quantitative data with a mathematical method in order to provide clear and robust clinical parameters and to allow outcome prediction [2]. The idea of radiomics is that the quantitative variables are more sensitively correlated with various clinical end-points compared with qualitative radiologic and clinical data [3]. Radiomic variables offer outstanding benefits over qualitative imaging assessment, since this is clearly limited by the resolution of radiologist’ eyes. A radiomic information extension can be obtained by adding genomics data (radiogenomics); in fact, genomic markers such as microRNA expression, have been shown associated with treatment response, metastatic spread and prognosis that could offer personalized and precision medicine [4,5,6]. Radiogenomics could perform patient selection for different cancer therapy, predict therapy, address potential therapy resistance (chemotherapy and/or radiation-therapy) and select patients with poor prognosis [3,7]. Various biomarkers have been individuated for advanced colorectal cancer (CRC) chemotherapy such as RAS and BRAF mutation status and microsatellite instability (MSI) status. RAS mutations are predictive of resistance towards anti-EGFR monoclonal antibodies [4,7]. BRAF mutant patients are correlated with a worse prognosis [4,7]. MSI-high status showed the immunotherapy efficacy in chemorefractory patients [4,7]. Moreover, based on literature results, RAS mutation (KRAS and NRAS) analysis is significant for anti-EGFR therapy selection and is deemed mandatory before beginning treatment in advanced CRC. In addition, CRC with wild-type RAS status is not always sensitive to anti-EGFR antibodies due to the less frequent mutations in the EGFR signaling pathway. BRAF-mutant CRC has a poor prognosis due to lower chemotherapy sensitivity and to clinical conditions that seriously affect the patients performance status [8,9]. A recent pooled analysis suggested that RAS mutation (including KRAS and NRAS) prevalence in metastatic CRC patients was about 55.9% [10].

The possibility to correlate radiomic parameters to RAS status offers notable advantages over qualitative imaging assessment, allowing one to tailor cancer therapy to the patient, to predict response to treatment, to dsitinguish favorable subsets of patients from those with poor prognosis, to select patients that may benefit of surgical treatment. In the present study, we assessed the association of RAS mutation status and radiomics derived data by Contrast Enhanced Magnetic Resonance Imaging (CE-MRI) in liver metastases by CRC. We performed the evaluation extracting by CE-MRI both texture and morphological metrics in a 3D setting.

## 2. Materials and Methods

### 2.1. Dataset Characteristics

All protocols were carried out in accordance with relevant guidelines and regulations. National Cancer Institute of Naples Ethical Committee board accepted this retrospective study. Patient informed consent was renounced.

Radiological databases were interrogated from 7 January 2018 to 17 December 2020 in order to select patients with liver metastases and underwent MR study and hepatic resection. The inclusion criteria were: (1) patients who had liver metastases with pathological proof; (2) patients with MR imaging at baseline before starting any chemotherapy treatment (for details on the chemotherapy regimen we refer to [11]); (3) availability of MR images of high quality. The exclusion criteria were: (1) discordance among the imaging diagnosis and the pathologically ones, (2) no baseline MR images and (c) no contrast MR images. The external validation patient dataset was provided by the University of Molise.

In total, 90 patients with pathologically confirmed liver metastases were found. Among them, 14 patients were excluded for the following reasons: (a) eight patients had no available diagnostic quality MR study images; (b) six patients had no contrast studies. Therefore, 76 patients (36 women and 40 men; 59 years of mean age and 36–80 years as range) with 130 liver metastases were included in the analysis. The validation cohort consisted of a total of 24 patients among 76 patients.

All liver metastases with confirmed RAS mutation status (KRAS and NRAS) were analyzed (among them 65 metastases with RAS mutation). The prevalence of RAS mutation frequency in this study agreed with the value for metastatic CRC patients reported in [10]. In our population there were patients with multiple liver lesions, however there were no patients with metastasis with different RAS mutation status. A consensus between radiologist and pathologist was considered to assess the correspondence between analyzed lesions.

Median time between magnetic resonance imagery acquisition and surgical resection was 12 days (range 7–21 days). The characteristics of the patients and their metastases are summarized in Table 1.

### 2.2. MR Imaging Protocol

MR examinations were performed with two 1.5 T MR scanners: a Magnetom Symphony (Siemens, Erlangen, Germany) and Magnetom Aera (Siemens) equipped with an 8-element body and phased array coils. The MRI study included basal images before intravenous (IV) contrast agent (CA) injection and dynamic sequences obtained after IV CA injection. Trufisp T2-weighted free breathing sequence was used for baseline images before IV CA injection while volumetric interpolated breath-hold examination (VIBE) T1-weighted SPAIR sequence was used to acquire dynamic images after IV CA injection with controlled respiration. As liver-specific CA, the Gd-EOB-BPTA (Primovist, Bayer Schering Pharma, Berlin, Germany) was employed. All patients received 0.1 mL/kg of Gd-EOB-BPTA by means of a power injector (Spectris Solaris^®^ EP MR, MEDRAD Inc., Indianola, IA, USA) at an infusion rate of 2 mL/s. Sequence parameters details of were reported in Table 2 as previously described in [12,13].

**Figure 1:**
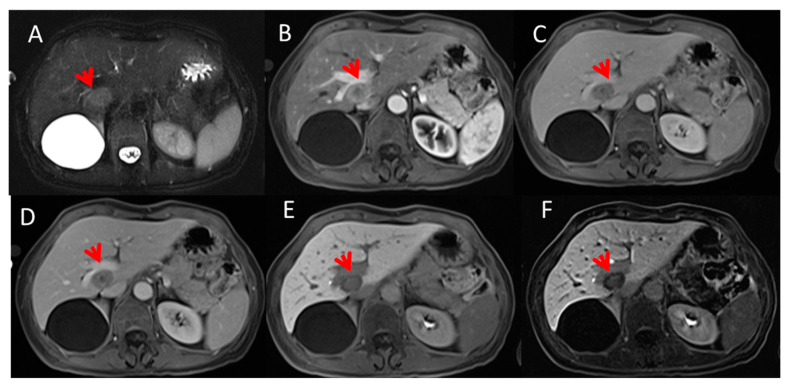
63 year-old man with rectal cancer. Liver metastasis on I hepatic segment. In (**A**) (TRUFISP T2-W fat sat) the lesion (arrow) is hyperintense. Contrast study with Gd-EOB-DTPA: the lesion (arrow) shows a peripheral rim enhancement with a hypointense core, in arterial phase (**B**). In the portal (**C**) and transitional phase (**D**), the lesion is hypointense. In the HPB phase (**E**,**F**), the lesion is hypointense, with a target appearance.

Figure 1 displays MR images with hepato-specific contrast of a case of liver metastasis with RAS mutation; by simple qualitative visual evaluation of the magnetic resonance images it was not possible to identify the RAS mutation state.

### 2.3. Data Analysis

Manual slice by slice segmentation was performed by two radiologists with 15 years of experience on MR liver images using an in-house program realized with Matlab R2007a (MathWorks, Natick, USA). The segmentation was performed on each phase of VIBE T1-W images. The metrics were obtained for each phase and then were calculated the median values of both texture and morphological parameters. For patients with multiple liver lesions, each metastasis was segmented: multiple nodules in the same patient range among 2–15 metastases (Table 1). Radiomics analysis were performed blinded to the clinical and pathological data. Moreover, radiomics analysis were performed on baseline MR before any chemotherapy treatment that patients have undergone. No registration techniques to reduce movements artefacts were applied, however the use of median value of extracted metrics on segmented volume of interest (VOI) allows to reduce the influence by artefacts.

#### 2.3.1. Texture Features

We considered a features set including 48 texture features. Texture features were obtained from VOIs manually segmented by CE-MRI for each time and then considering the median value among nine series. The texture metrics included both first order features (mean, mode, median, standard deviation (std), median absolute deviation (MAD), range, kurtosis, skewness, and the interquartile range (IQR)) and second order features. For these letters was used the “Texture Toolbox” of MATLAB. The texture analysis package implements wavelet band-pass filtering, isotropic resampling, discretization length corrections and different quantization tools [14]. An exhaustive explanation has been provided in Vallières et al. [14]. The toolbox can be downloaded at https://it.mathworks.com/matlabcentral/fileexchange/51948-radiomics. The package of texture features is adherent to Image biomarker standardization initiative [15]. A detailed description has been provided in the Supplementary File S1.

#### 2.3.2. Morphological Features

We considered a features set including 15 morphological features [16,17,18] calculated using an in-house MATLAB script. A detailed description of the morphological features has been provided in Table 3.

### 2.4. Statistical Analysis

Statistical analysis includes both univariate and multivariate approaches performed considering a per-patient analysis. 

#### 2.4.1. Univariate Analysis 

The calculation of inter-observer variability between two readers and the evaluation of unstable features were performed. The assessment of observer variability was performed by calculating the intraclass correlation coefficient [19].

For each metric, median, range or confident interval (CI) values were calculated on segmented VOI. Receiver operating characteristic (ROC) analyses were obtained and the optimal cut-off value for each feature was calculated using the Youden index. Area under ROC curve (AUC), sensitivity (SEN), specificity (SPEC), positive predictive value (PPV), negative predictive value (NPV) and accuracy (ACC) were calculated using the optimal threshold identified with the Youden index. Non-parametric Wilcoxon-Mann-Whitney U test for two-groups comparisons was used. 

A *p* value < 0.05 was considered as significant. However, false discovery rate (FDR) adjustment according to Benjamini and Hochberg [20] for multiple testing was considered. The statistical analysis was performed using the Statistics Toolbox of Matlab R2007a.

#### 2.4.2. Multivariate Analysis

Pattern recognition methods (linear discrimination analysis (LDA), support vector machine (SVM), k-nearest neighbors (KNN), artificial neural network (NNET), and decision tree (DT)) were considered to assess the diagnostic accuracy in a multivariate analysis [21]. The best model was chosen considering the highest area under ROC curve and highest accuracy.

The analysis was made before and after a feature selection method. The least absolute shrinkage and selection operator (LASSO) method was used to detect the robust features [22]. A 10-fold cross-validation was used to select the optimal regularization parameter alpha in the LASSO method, as the average of mean square error of each patient was the smallest. Considering the identified optimal alpha, only the features having nonzero coefficient were reserved.

A 10-k fold cross validation approach was used to individuate the best classifier on the training set; therefore, median and 95% confidence interval values of AUC, accuracy, sensitivity and specificity were calculated. However, an external validation cohort was used to validate the findings of the best classifier. Multivariate analysis was performed using the statistics and Machine Learning Toolbox of Matlab R2007a.

## 3. Results

### 3.1. Univariate Analysis Findings

There were 17 stable features (identified as intraclass correlation coefficient value ≥ 0.8) were (14 texture features and three morphological ones): variance, contrast, dissimilarity, short run emphasis (SRE), run-length nonuniformity (RLN), run percentage (RP), small zone emphasis (SZE), zone-size non-uniformity (ZSN), zone percentage (ZP), small zone low gray-level emphasis (SZLGE), gray-level variance (GLV), coarseness, entropy, strength, circularity, compactness, convexity. The median value of intraclass correlation coefficients for stable features was 0.9 (range 0.85–0.96). The size of the lesion did not affect the stable metrics (*p*-value > 0.05 at the Wilcoxon-Mann-Whitney U test performed between the groups obtained by dividing patients with lesions <2 cm and patients with lesions ≥2 cm).

**Figure 2:**
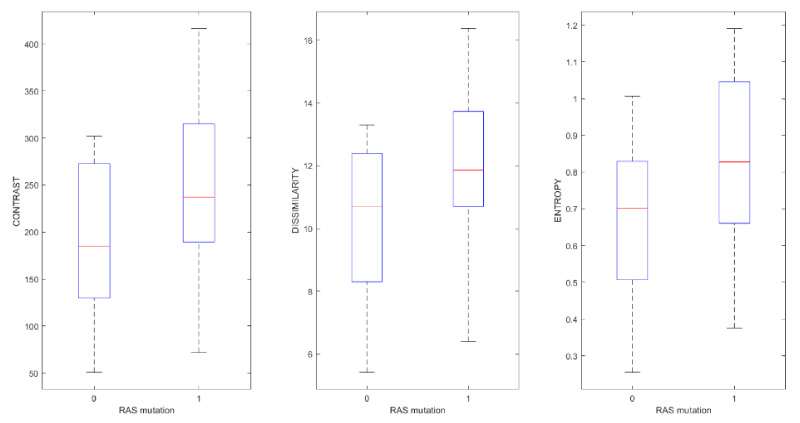
Boxplot for texture features to detect RAS mutation.

Table 4 reports median and range values for the radiomic metrics that obtained significant results by Wilcoxon-Mann-Whitney U test to differentiate patients with and without RAS mutation. Significant results were obtained for the following texture parameters: contrast, dissimilarity and entropy (Figure 2 and Table 5), also considering the FDR adjustment. No morphological feature had significant results to differentiate patients with and without RAS mutation.

**Figure 3:**
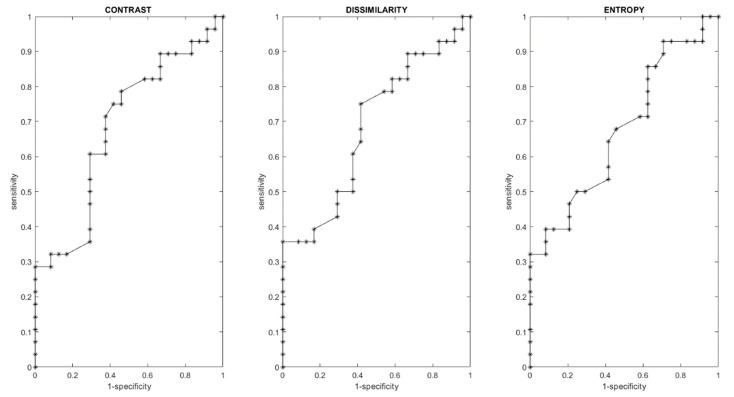
ROC curves for significant textual features to detect RAS mutation.

Figure 2 shows a boxplot for the significant texture features to detect RAS mutations. Table 5 reports the diagnostic accuracy for the individuated significant texture features to detect RAS mutation. Figure 3 shows the ROC curves for significant textual features to detect RAS mutation. 

The results showed that using a univariate analysis was not possible to discriminate accurately the RAS mutation status.

### 3.2. Multivariate Analysis Findings

#### 3.2.1. Traing Set Results 

The training set included 52 patients 28 with RAS mutation and 24 wild type. Considering stable texture and morphological metrics tested with pattern recognition approaches, the best performance to detect RAS mutation was reached using a SVM with AUC of 0.79 (0.70–0.85 95% confidence interval (CI)), an accuracy of 76.1% (72–82% 95% CI) with 74.2% (70–84% 95% CI) of sensitivity and 78.0% (75–89% 95% CI) of specificity.

Considering LASSO results, the robust features to use as predictors were contrast, dissimilarity, RLN, RP, and entropy. 

**Figure 4:**
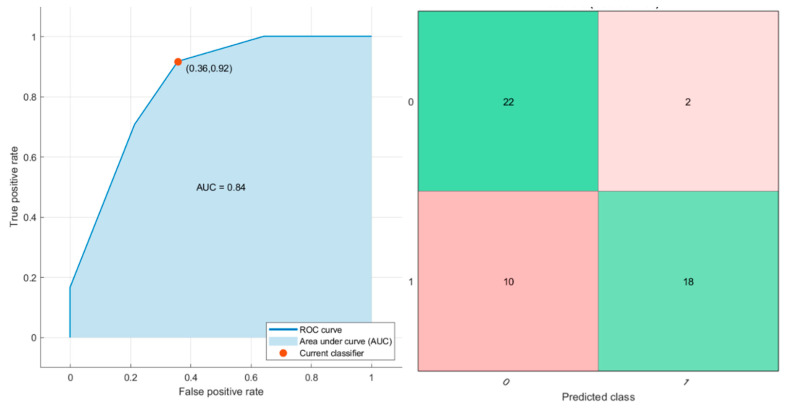
ROC curve, confusion matrix and graphical view of the decision tree considering the robust features by LASSO method to use as predictors.

A KNN trained with these predictors achieved the best results with an AUC of 0.84 (0.8–0.91 95% CI), an accuracy of 76.9% (71–82% 95% CI) with 90.0% (85–99% 95% CI) of sensitivity and 67.8% (60–75% 95% CI) of specificity (Figure 4). 

#### 3.2.2. External Validation Results 

External validation cohort included 24 patients, 13 with RAS mutation and 11 wild type. Considering all stable texture and morphological metrics, the SVM obtained an accuracy of 79.2% with 83.3% of sensitivity and 75.0% of specificity (10 positive trues, nine negative trues, three false positive and two false negative). Considering the robust features, the KNN obtained an accuracy of 87.5% with 91.7% of sensitivity and 83.3% of specificity (11 positive trues, 10 negative trues, two false positive and one false negative).

## 4. Discussion

Literature data has reported the potential role of radiomics to realize personalized medicine in different diseases such as cancer and to select more appropriate therapy correlated to the different tumour subtype [18]. Literature data underlines the role of RAS mutations as a strong prognostic and predictive biomarker in patients subjected to hepatic resection for CRLM [19]. RAS mutations were strongly associated with worse overall survival (OS) and recurrence-free survival (RFS) in patients with CRLM [23]. The possibility to correlate radiomic parameters to RAS status offers notable advantages over qualitative imaging assessment allowing one to tailor cancer therapy at the patient, to predict response to treatment, to detect favorable subsets of patients from those with poor prognosis and to select patients that may benefit of surgical treatment.

In this study we assessed radiomics derived data by contrast enhanced magnetic resonance imaging in association with RAS mutation status in liver metastases, showing significant results exclusively for texture parameters. At the best of our knowledge, this is the first paper that evaluates the correlation between radiomics data and RAS mutations in liver metastases using features extracted by contrast enhanced magnetic resonance imaging.

However, using univariate analysis, the accuracy achieved was not satisfactory to stratify RAS mutation status. Promising, instead, were in this study the results of multivariate analysis. In fact, considering a per-patient multivariate analysis and classification approaches, a KNN exclusively with texture parameters as predictors achieved the best results with an AUC of 0.84, an accuracy of 76.9% with 90.0% of sensitivity and 67.8% of specificity on training set. The KNN method, on the test set, obtained an accuracy of 87.5% with 91.7% of sensitivity and 83.3% of specificity on external validation cohort.

Several studies have assessed the role of radiomics parameters as a precision medicine tool that may affect treatment strategies. Zhang et al. [24] assessed radiomics parameters extracted by contrast enhanced MRI T1 weighted images and T2 weighted morphological MR images as prognostic factors in patients with advanced nasopharyngeal carcinoma (NPC), showing a significant improvement over the TNM staging system in terms of progression-free survival (PFS) evaluation. Cui et al. [25] assessed a radiomics model of multiparametric MRI features and clinical features to predict a pathological complete response (pCR) in patients with locally advanced rectal cancer (LARC) after neoadjuvant chemo-radiotherapy (CRT), demonstrating that the radiomics model can predict pCR and can select patients for a “wait-and-see” strategy. Although liver metastases are a leading cause of colorectal cancer, the molecular genetic basis of the advanced disease stages remains poorly understood. Whether the metastatic lesions are genetically homogeneous or heterogeneous may determine the response to therapy [26,27,28,29]. Sung et al. [28] investigated whether synchronously-occurring, multi-focal colon cancer liver metastases were of multi-clonal origin by using genome-scale microarray analysis. They concluded that genetic profiling of multiple liver metastases using genome scale profiling suggests that colon cancer metastases are muti-clonal in origin.

The current study had some limits: the monocentric nature of the study; the small size of the patient sample, the time consuming manual segmentation of lesions, that fact that in this study we did not consider combining radiomics features with other clinical prognostic factors that can be considered as a future end-point and the research is retrospective. Therefore, further prospective multicenter analyses including more patients are needed to validate the prognostic significance of these results.

## 5. Conclusions

Radiomics with texture parameters could add value to qualitative assessment of MR studies and with better results compared to morphological metrics, providing individualized evaluation of CRLM. Texture parameters derived by CE-MRI and combined using multivariate analysis and patter recognition approaches could allow stratifying the patients according to RAS mutation status.

## Figures and Tables

**Table 1:** Characteristics of the study population.

Patient Description	Numbers (%)/Range
Gender	Men 40 (52.6%)
	Women 36 (47.4%)
Age	59 y; range: 36–80 y
**Primary cancer site**	
Colon	41 (53.9%)
Rectum	35 (46.1%)
**Hepatic metastases description**	
Patients with single nodule	44 (57.9%)
Patients with multiple nodules	32 (42.1%)/range: 2–15 metastases
Nodule size (mm)	mean size 34.9 mm; range 18–54 mm
**RAS mutation**	41 (53.9%)
Wild Type	35 (46.1%)

**Table 2:** MR Sequence parameters.

Sequence	Orientation	TR/TE/FA (ms/ms/deg.)	AT (min.)	Acquisition Matrix	Slice Thickness/Gap (mm)	Fat Suppression
TRUFISPT2-W	Coronal	4.30/2.15/80	0.46	512 × 512	4/0	without
VIBET1-W	Axial	4.80/1.76/12	0.18	320 × 260	3/0	with (SPAIR)

Note. W = Weighted, TR = Repetition time, TE = Echo time, FA = Flip angle, AT = Acquisition time, SPAIR = Spectral Adiabatic Inversion Recovery, VIBE = Volumetric interpolated breath hold examination.

**Table 3:** Morphological parameters description and formula.

Features	Description	Formula
Radial length average	Radial lengths average quantification obtained measuring for each point on the ROI border the Euclidean distance from the mass centroid	$R_{\mathrm{avg}}\frac{1}{b_{\mathrm{ROI}}}\overset{b_{\mathrm{ROI}\;}}{\underset{j=1}{\sum }}R_{j}$ $b_{\mathrm{ROI}}$ the number of boundary voxels in the ROI
Radial length entropy	Quantification of the entropy of radial length	$-\overset{b_{\mathrm{ROI}}}{\underset{j=1}{\sum }}P_{{j\;\log}_{\begin{array}{c} 2\; \end{array}}}\left(P_{j}\right)$; $P_{j}=\frac{R_{j}}{\sum R_{j}}$
Volume	Quantification of the entire volume of segmented lesion	$V=n_{\mathrm{ROI}}\mathrm{dxdydz}$ $n_{\mathrm{ROI}\;}$be the total number of voxel in region d_x_ d_y_ d_z_ represent size of voxel
Surface	Quantification of the surface of segmented lesion	$\;\underset{x}{\sum }\underset{y}{\sum }\underset{z}{\sum }b_{\mathrm{ROI}}\left(x,y,z\right)v_{\mathrm{size}}{\mathrm{slice}}_{\mathrm{th}}$ $v_{\mathrm{size}}$ is the voxel size, ${\mathrm{slice}}_{\mathrm{th}}$ is the slice thickness
Circularity	3D evaluation of the lesion conformity to a sphere	$\frac{V_{\mathrm{sph}}\left(\mathrm{effective}\;\mathrm{diameter}\right)}{V}$ $\mathrm{effective}\;\mathrm{diameter}=2\sqrt[{3}]{\frac{3V}{4\pi }}$
Compactness	Evaluation of the relationship between surface and volume	$\frac{S^{2}}{V}$
Rectangularity	Evaluation of the lesion volume respect to the smallest rectangular that would contain it	$\frac{V}{V_{\mathrm{rec}}}$
Roughness	Quantification of the roughness of the lesion	$\frac{\sqrt[{4}]{\frac{1}{n_{\mathrm{ROI}}}\sum_{j=1}^{n_{\mathrm{kROI}}}{\left(r_{j}-R_{\mathrm{avg}}\right)}^{4\;}}-\sqrt{\frac{1}{n_{\mathrm{ROI}}}\sum_{j=1}^{n_{\mathrm{kROI}}}{\left(r_{j}-R_{\mathrm{avg}}\right)}^{2}}}{R_{\mathrm{avg}}}$
Smoothness	Calculation of the lesion contours irregularities	$\underset{b\;\in R,\;i\;\in \left\{0,\;\ldots ,\;N-1\right\}\;}{\sum }D\left(b^{i}\right)$ $D\left(b^{i}\right)=\left|∥b^{i\;}-c\text{¯}\|-\frac{\|b^{i-1\;}-c\text{¯}\|+\|b^{i+1\;}-c\text{¯}\|}{2}\right|$ c¯ represents the center of mass of the lesion in two dimensions and $b^{i}$ the points on the border of the lesion
Irregularity	Calculation of the surface roughness of the lesion	$1-\frac{V_{\mathrm{sph}}\left(\mathrm{effective}\;\mathrm{diameter}\right)}{V}$
Sphericity	Evaluation of the Sphericity of the lesion	Ratio between the average radial length and the standard deviation of the rays
Convexity	Measure of the ratio between the minimum area with convex curvature that connects the voxels to the edge with respect to the original ROI area	$\frac{R_{\mathrm{avg}}}{R_{\mathrm{sd}}}$ where R_sd_ represents the standard deviation of the radial lengths
Eccentricity	Measure of the ratio of the larger rope and the largest among the orthogonal ropes	= (lesion largest diameter)/(the largest diameter orthogonal to the previous one)
Elogation	Estimation of how much the lesion is pronounced along one direction than along the other	= (length)/(width) of the smallest rectangle containing the lesion averaged per each slice in three orthogonal directions

Note. ROI = region of interest.

**Table 4:** Median and range values for the radiomic extracted metrics.

Patient	Value	CONTRAST	DISSIMILARITY	ENTROPY
Without RAS mutation	Median	179.42	10.36	0.70
	Range (minimum-maximum)	251.48	7.88	0.75
With RAS mutation	Median	231.71	11.72	0.90
	Range (minimum-maximum)	453.41	10.83	1.02
Total	Median	212.26	10.93	0.75
	Range (minimum-maximum)	474.47	11.81	1.12

**Table 5:** Diagnostic accuracy for the individuated significant features to detect RAS mutation.

Feature	AUC (95% Confidence Interval)	SEN	SPEC	PPV	NPV	ACC	CUT-OFF	*p* Value
CONTRAST	0.69 (0.47–0.75)	0.71	0.63	0.69	0.65	0.67	192.86	**0.00**
DISSIMILARITY	0.69 (0.46–0.76)	0.36	1.00	1.00	0.57	0.65	13.30	**0.00**
ENTROPY	0.68 (0.42–0.72)	0.32	1.00	1.00	0.56	0.63	1.01	**0.00**

Note. AUC = are under curve; SEN = sensitivity; SPEC = specificity; PPV = positive predictive value; NPV = negative predictive value; ACC = accuracy; *p* Value obtained by Wilcoxon-Mann-Whitney U test. In bold were reported the significant p values considering FDR adjustment.

## Data Availability

The data presented in this study are available in the manuscript.

## Supplementary Materials

The following are available online at https://www.mdpi.com/2072-6694/13/3/453/s1, Supplementary File S1: Definition of textural features.
