# Supplementary material for: Radiomics-Derived Data by Contrast Enhanced Magnetic Resonance in RAS Mutations Detection in Colorectal Liver Metastases
Source: Cancers (Basel). 2021 Jan 25;13(3):453. doi: 10.3390/cancers13030453 (PMC7865653; doi:10.3390/cancers13030453)
Supplement: Supplementary file 1 [file cancers-13-00453-s001.pdf]

Appendix A: Definition of textural features

First-order gray-level statistics

First order gray-level statistics describe the distribution of gray-values within the volume. Let X denote the 3-D image matrix with N voxels, P the first order histogram, $P(i)$ the fraction of voxels with intensity level i and Nl the number of discrete intensity levels.

- **Mean**, the mean gray-level of X.

$$mean = \frac{1}{N}\sum_{i=1}^{N} X(i)$$

- **Mode**, the most frequent element(s) of array X.
- **Median**, the sample median of X, or the 50th percentile of X.
- **Standard deviation (STD)**

$$STD = \left(\frac{1}{N-1}\sum_{i=1}^{N}(X(i) - \bar{X})^2\right)^{1/2}$$

- **Mean Absolute Deviation (MAD)**, the mean of the absolute deviation of all voxel intensities around the mean intensity value.

$$MAD = \frac{1}{N}\sum_{i=1}^{N}|X(i) - \bar{X}|$$

- **Range**, the range of intensity values of X.

$$range = \max(X) - \min(X)$$

  where $\max(X)$ is the maximum intensity value of X and $\min(X)$ is the minimum intensity value of X.

- **Interquartile range (IQR)**, the interquartile range is defined as the 75th minus the 25th percentile of X.
- **Kurtosis**

$$kurtosis = \frac{\frac{1}{N}\sum_{i=1}^{N}(X(i) - \bar{X})^4}{\left(\sqrt{\frac{1}{N}\sum_{i=1}^{N}(X(i) - \bar{X})^2}\right)^2}$$

where $\bar{X}$ is the mean of X.

- **Variance,** Variance is the square of the standard deviation.

$$variance = \frac{1}{N-1}\sum_{i=1}^{N}(X(i) - \bar{X})^2$$

where $\bar{X}$ is the mean of X.

- **Skewness**

$$skewness = \frac{\frac{1}{N}\sum_{i=1}^{N}(X(i) - \bar{X})^3}{\left(\sqrt{\frac{1}{N}\sum_{i=1}^{N}(X(i) - \bar{X})^2}\right)^3}$$

where $\bar{X}$ is the mean of X.

Gray-Level Co-Occurrence Matrix (GLCM) [1-3]

A normalized GLCM is defined as $P(i,j;\delta,\alpha)$, a metric with size $N_g \times N_g$ describing the second-order joint probability function of an image, where the (i,j)th element represents the number of times the combination of intensity levels i and j occur in two pixels in the image, that are separated by a distance of δ pixels in direction α, and N_g is the maximum discrete intensity level in the image. Let:

- $P(i,j)$ be the normalized (i.e. $\sum P(i,j) = 1$) co-occurrence matrix, generalized for any δ and α,
- $p_x(i) = \sum_{j=1}^{N_g} P(i,j)$,
- $p_y(j) = \sum_{i=1}^{N_g} P(i,j)$,
- μ_x be the mean of p_x, where $\mu_x = \sum_{i=1}^{N_g}\sum_{j=1}^{N_g} iP(i,j)$,
- μ_y be the mean of p_y, where $\mu_y = \sum_{i=1}^{N_g}\sum_{j=1}^{N_g} jP(i,j)$,
- σ_x be the standard deviation of p_x, where $\sigma_x = \sum_{i=1}^{N_g}\sum_{j=1}^{N_g} P(i,j)(i-\mu_x)^2$,
- σ_y be the standard deviation of p_y, where $\sigma_y = \sum_{i=1}^{N_g}\sum_{j=1}^{N_g} P(i,j)(j-\mu_y)^2$.

- **Energy**

$$energy = \sum_{i=1}^{N_g}\sum_{j=1}^{N_g}[P(i,j)]^2$$

- **Contrast**

$$contrast = \sum_{i=1}^{N_g}\sum_{j=1}^{N_g}|i-j|^2 P(i,j)$$

- **Entropy**

$$entropy = -\sum_{i=1}^{N_g}\sum_{j=1}^{N_g}P(i,j)log_2\,[P(i,j)]$$

- **Homogeneity**

$$homogeneity = \sum_{i=1}^{N_g}\sum_{j=1}^{N_g}\frac{P(i,j)}{1+\ |i-j|}$$

- **Correlation**

$$correlation = \frac{\sum_{i=1}^{N_g}\sum_{j=1}^{N_g} ijP(i,j) - \ \mu_x\mu_y}{\sigma_x\sigma_y}$$

- **Sum Average**

$$sum\ average = \frac{1}{N_g \times N_g}\sum_{i=1}^{N_g}\sum_{j=1}^{N_g}[iP(i,j) + jP(i,j)]$$

- **Dissimilarity**

$$dissimilarity = \sum_{i=1}^{N_g}\sum_{j=1}^{N_g}|i-j|P(i,j)$$

- **Autocorrelation**

$$autocorrelation = \sum_{i=1}^{N_g}\sum_{j=1}^{N_g} ijP(i,j)$$

Gray-Level Run-Length Matrix (GLRLM) [4-7]

Run length metrics quantify gray level runs in an image. A gray level run is defined as the length in number of pixels, of consecutive pixels that have the same gray level value. In a gray level run length matrix $p(i,j|\theta)$, the (i,j)th element describes the number of times j a gray level i appears consecutively in the direction specified by θ. Let:

- $p(i,j)$ be the (i,j)th entry in the given run-length matrix p, generalized for any direction θ,
- N_g the number of discrete intensity values in the image,
- N_r the maximum run length,
- N_s the total numbers of runs, where $N_s = \sum_{i=1}^{N_g}\sum_{j=1}^{N_r} p(i,j)$
- p_r the sum distribution of the number of runs with run length j, where $p_r(j) = \sum_{i=1}^{N_g} p(i,j)$,
- p_g the sum distribution of the number of runs with run length i, where $p_g(i) = \sum_{j=1}^{N_r} p(i,j)$,
- N_p the number of voxels in the image, where $N_p = \sum_{j=1}^{N_r} jp_r$,
- μ_r the mean run length, where $\mu_r = \sum_{i=1}^{N_g}\sum_{j=1}^{N_r} jp_n(i,j)$,
- μ_g the mean gray level, where $\mu_g = \sum_{i=1}^{N_g}\sum_{j=1}^{N_r} ip_n(i,j)$.

- **Short Run Emphasis (SRE)**

$$SRE = \sum_{j=1}^{N_r} \frac{p_r}{j^2}$$

- **Long Run Emphasis (LRE)**

$$LRE = \sum_{j=1}^{N_r} j^2 p_r$$

- **Gray-Level Nonuniformity (GLN)**

$$GLN = \sum_{i=1}^{N_g} {p_g}^2$$

- **Run-Length Nonuniformity (RLN)**

$$RLN = \sum_{j=1}^{N_r} {p_r}^2$$

- **Run Percentage (RP)**

$$RP = \frac{N_s}{N_p}$$

- **Low Gray-Level Run Emphasis (LGRE)**

$$LGRE = \sum_{i=1}^{N_g} \frac{p_g}{i^2}$$

- **High Gray-Level Run Emphasis (HGRE)**

$$HGRE = \sum_{i=1}^{N_g} i^2 p_g$$

- **Short Run Low Gray-Level Emphasis (SRLGE)**

$$SRLGE = \sum_{i=1}^{N_g} \sum_{j=1}^{N_r} \frac{p(i,j)}{i^2 j^2}$$

- **Short Run High Gray-Level Emphasis (SRHGE)**

$$SRHGE = \sum_{i=1}^{N_g} \sum_{j=1}^{N_r} \frac{p(i,j) i^2}{j^2}$$

- **Long Run Low Gray-Level Emphasis (LRLGE)**

$$LRLGE = \sum_{i=1}^{N_g} \sum_{j=1}^{N_r} \frac{p(i,j) j^2}{i^2}$$

- **Long Run High Gray-Level Emphasis (LRHGE)**

$$LRHGE = \sum_{i=1}^{N_g} \sum_{j=1}^{N_r} p(i,j) i^2 j^2$$

- **Gray-Level Variance (GLV)**

$$GLV = \frac{1}{N_g \times N_r} \sum_{i=1}^{N_g} \sum_{j=1}^{N_r} \left(ip(i,j) - \mu_g\right)^2$$

- **Run-Length Variance (RLV)**

$$RLV = \frac{1}{N_g \times N_r} \sum_{i=1}^{N_g} \sum_{j=1}^{N_r} (jp(i,j) - \mu_r)^2$$

Gray-Level Size Zone Matrix (GLSZM) [4-7]

A gray level size-zone matrix describes the amount of homogeneous connected areas within the volume, of a certain size and intensity. The (i,j)th entry of the GLSZM $p(i,j)$ is the number of connected areas of gray-level (i.e. intensity value) i and size j. GLSZM features therefore describe homogeneous areas within the tumor volume, describing tumor heterogeneity at a regional scale [5]. Let:

- $p(i,j)$ be the (i,j)th entry in the given GLSZM p,
- N_g the number of discrete intensity values in the image,
- N_z the size of the largest, homogeneous region in the volume of interest,
- N_s the total number of homogeneous regions (zones), where $N_s = \sum_{i=1}^{N_g} \sum_{j=1}^{N_z} p(i,j)$
- p_z the sum distribution of the number of zones with size j, where $p_z(j) = \sum_{i=1}^{N_g} p(i,j)$,
- p_g the sum distribution of the number of zones with gray level i, where $p_g(i) = \sum_{j=1}^{N_z} p(i,j)$,
- N_p the number of voxels in the image, where $N_p = \sum_{j=1}^{N_z} jp_r$,
- μ_r the mean zone size, where $\mu_r = \sum_{i=1}^{N_g} \sum_{j=1}^{N_z} jp(i,j|\theta)$,
- μ_g the mean gray level, where $\mu_g = \sum_{i=1}^{N_g} \sum_{j=1}^{N_z} ip(i,j|\theta)$.

- **Small Zone Emphasis (SZE)**

$$SZE = \sum_{j=1}^{N_z} \frac{p_z}{j^2}$$

- **Large Zone Emphasis (LZE)**

$$LZE = \sum_{j=1}^{N_z} j^2 p_z$$

- **Gray-Level Non-uniformity (GLN)**

$$GLN = \sum_{i=1}^{N_g} {p_g}^2$$

- **Zone-Size Non-uniformity (ZSN)**

$$ZSN = \sum_{i=1}^{N_g} {p_z}^2$$

- **Zone Percentage (ZP)**

$$ZP = \frac{N_s}{N_p}$$

- **Low Gray-Level Zone Emphasis (LGZE)**

$$LGZE = \sum_{i=1}^{N_g} \frac{p_g}{i^2}$$

- **High Gray-Level Zone Emphasis (HGZE)**

$$HGZE = \sum_{i=1}^{N_g} i^2 p_g$$

- **Small Zone Low Gray-Level Emphasis (SZLGE)**

$$SZLGE = \sum_{i=1}^{N_g} \sum_{j=1}^{N_z} \frac{p(i,j)}{i^2 j^2}$$

- **Small Zone High Gray-Level Emphasis (SZHGE)**

$$SZHGE = \sum_{i=1}^{N_g} \sum_{j=1}^{N_z} \frac{p(i,j) i^2}{j^2}$$

- **Large Zone Low Gray-Level Emphasis (LZLGE)**

$$LZLGE = \sum_{i=1}^{N_g} \sum_{j=1}^{N_z} \frac{p(i,j) j^2}{i^2}$$

- **Large Zone High Gray-Level Emphasis (LZHGE)**

$$LZHGE = \sum_{i=1}^{N_g}\sum_{j=1}^{N_z} p(i,j)j^2 i^2$$

- **Gray-Level Variance (GLV)**

$$GLV = \frac{1}{N_g \times N_z}\sum_{i=1}^{N_g}\sum_{j=1}^{N_z}\left(ip(i,j) - \mu_g\right)^2$$

- **Zone-Size Variance (ZSV)**

$$ZSV = \frac{1}{N_g \times N_z}\sum_{i=1}^{N_g}\sum_{j=1}^{N_z}(jp(i,j) - \mu_z)^2$$

Neighborhood gray tone difference matrix (NGTDM) [8]

The ith entry of the NGTDM $s(i|d)$ is the sum of gray level differences of voxels with intensity i and the average intensity A_i of their neighboring voxels within a distance d. Let:

- n_i be the number of voxels with gray level i,
- $N = \sum n_i$, the total number of voxels,
- $s(i) = \begin{cases} \sum_{n_i} |i - A_i| & for\ n_i > 0 \\ 0 & otherwise \end{cases}$, generalized for any distance d,
- N_g be the maximum discrete intensity level in the image,
- $p(i) = \frac{n_i}{N}$, the probability of gray level i,
- N_p, the total number of gray levels present in the image.

- **Coarseness**

$$coarseness = \left[\varepsilon + \sum_{n=1}^{N_g} p(i)s(i)\right]^{-1}$$

where ε is a small number to prevent coarseness becoming infinite.

- **Contrast**

$$contrast = \left(\frac{1}{N_p(1-N_p)}\sum_{i=1}^{N_g}\sum_{j=1}^{N_g} p(i)p(j)(i-j)^2\right)\left(\frac{1}{N}\sum_{i=1}^{N_g} s(i)\right)$$

- **Busyness**

$$busyness = \frac{\sum_{i=1}^{N_g} p(i)s(i)}{\sum_{i=1}^{N_g}\sum_{j=1}^{N_g}|ip(i) - jp(j)|}, \qquad p(i) \neq 0, p(j) \neq 0$$

- **Complexity**

$$complexity = \sum_{i=1}^{N_g}\sum_{j=1}^{N_g}|i-j|\frac{p(i)s(i) + p(j)s(j)}{N\big(p(i) + p(j)\big)}, \qquad p(i) \neq 0, p(j) \neq 0$$

- **Strength**

$$strength = \frac{\sum_{i=1}^{N_g}\sum_{j=1}^{N_g}[p(i) + p(j)](i-j)^2}{\varepsilon + \sum_{n=1}^{N_g} s(i)}, \qquad p(i) \neq 0, p(j) \neq 0$$

where ε is a small number to prevent strength becoming infinite.

Table 2.3 Texture features list.

Feature Name	Symbol	Reference
**1st order gray-level statistics**		-
- Mean	-	
- Mode	-	
- Median	-	
- Standard Deviation	STD	
- Median Absolute Deviation	MAD	
- Range	-	
- Kurtosis	-	
- Interquartile Range	IQR	
- Variance	-	
- Skewness	-	
**Gray-Level Co-occurrence Matrix (GLCM)**		Haralick et al. [3]
- Energy	-	
- Contrast	-	
- Entropy	-	
- Homogeneity	-	
- Correlation	-	
- Sum Average	-	
- Dissimilarity	-	
- Autocorrelation	-	
**Gray-Level Run-Length Matrix (GLRLM)**		Galloway [4]
- Short Run Emphasis	SRE	Chu et al. [5]
- Long Run Emphasis	LRE	Dasarathy and Holder
- Gray-Level Nonuniformity	GLN_GLRLM	[6]
- Run-Length Nonuniformity	RLN	Thibault et al. [7]
- Run Percentage	RP	
- Low Gray-Level Run Emphasis	LGRE	
- High Gray-Level Emphasis	HGRE	
- Short Run Low Gray-Level Emphasis	SRLGE	
- Short Run High Gray-Level Emphasis	SRHGE	
- Long Run Low Gray-Level Emphasis	LRLGE	
- Long Run High Gray-Level Emphasis	LRHGE	
- Gray-Level Variance	GLV_GLRLM	
- Run-Length Variance	RLV	
**Gray-Level Size Zone Matrix (GLSZM)**		Galloway [4]
- Small Zone Emphasis	SZE	Chu et al. [5]
- Large Zone Emphasis	LZE	Dasarathy and Holder
- Gray-Level Non-uniformity	GLSZM_GLN	[6]
- Zone-Size Non-uniformity	ZSN	Thibault et al. [7]
- Zone Percentage (ZP)	ZP	
- Low Gray-Level Zone Emphasis	LGZE	
- High Gray-Level Zone Emphasis	HGZE	
- Small Zone Low Gray-Level Emphasis	SZLGE	
- Small Zone High Gray-Level Emphasis	SZHGE	
- Large Zone Low Gray-Level Emphasis	LZLGE	
- Large Zone High Gray-Level Emphasis	LZHGE	
- Gray-Level Variance	GLV_GLSZM	
- Zone-Size Variance	ZSV	
**Neighbourhood gray-tone difference matrix (NGTDM)**		Amadasun and King
- Coarseness	-	[8]
- Busyness	-	
- Complexity	-	
- Strength	-	

References

[1] Thibault G. Indices de formes et de textures: de la 2D vers la 3D. Application au classement de noyaux de cellules. PhD Thesis, Université AIX-Marseille, 2009 Jun 18.

[2] Aerts H.J.W.L., Velazquez, E.R., Leijenaar, R.T.H., Parmar C., Grossmann P., Carvalho S., Bussink J., Monshouwer R., Haibe-Kains B., Rietveld D., Hoebers F., Rietbergen M.M., Leemans C.R., Dekker A., Quackenbush J., Gillies R.J., Lambin P. Decoding tumour phenotype by non-invasive imaging using a quantitative radiomics approach. Nature Communication, 2014 June 03; 5(1):4006. doi: 10.1038/ncomms5006

[3] Haralick R.M., Shanmugam K., Dinstein I.H. Textural features for image classification. IEEE Transactions on Systems, Man and Cybernetics, 1973 Nov; SMC-3(6):610–621. doi: 10.1109/TSMC.1973.4309314

[4] Galloway M.M. Texture analysis using gray level run lengths. Computer Graphics and Image Processing, 1975 Jun; 4(2):172-9. doi: 10.1016/S0146-664X(75)80008-6

[5] Chu A., Sehgal C., Greenleaf J. Use of gray value distribution of run lengths for texture analysis. Pattern Recognition Letters, 1990 Jun; 11(6):415-9. doi: 10.1016/0167-8655(90)90112-F

[6] Dasarathy B., Holder E. Image characterizations based on joint gray level-run length distributions. Pattern Recognition Letters, 1991 Aug; 12(8):497-502. doi: 10.1016/0167-8655(91)80014-2

[7] Thibault G., Fertil B., Navarro C., Pereira S., Cau P., Levy N., Sequeira J., Mari J.L. Texture indexes and gray level size zone matrix: application to cell nuclei classification. Pattern Recognition and Information Processing, 2009.

[8] Amadasun M., King R. Textural features corresponding to textural properties. IEEE Transactions on Systems, Man, and Cybernetics, 1989 Sep/Oct; 19(5):1264-74. doi: 10.1109/21.44046
